# Protective factors for early initiation of breastfeeding among Brazilian nursing mothers

**DOI:** 10.3389/fped.2023.1203575

**Published:** 2023-06-09

**Authors:** Giovana Gaglianone Lemos, Taciana Maia de Sousa, Rafaela Cristina Vieira e Souza, Larissa Bueno Ferreira, Cristianny Miranda, Luana Caroline dos Santos

**Affiliations:** ^1^ Nutrition Department, Universidade Federal de Minas Gerais, Belo Horizonte, Brazil; ^2^ Nutrition Department, Universidade de Brasília, Brasília, Brazil

**Keywords:** breastfeeding, postpartum period, nursing mothers, child health, antenatal care

## Abstract

**Objectives:**

Evaluate the protective factors associated with early initiation of breastfeeding.

**Methods:**

Cross-sectional study conducted with Brazilian nursing mothers. Breastfeeding in the first hour of life and difficulty initiating breastfeeding in the birth room were adopted as outcome variables and associated with other maternal and child information. Poisson regression was conducted to synthesize the data.

**Results:**

Among 104 nursing mothers evaluated, 56.7% reported breastfeeding in the first hour of life and 43% had difficulty initiating breastfeeding in the birth room. There was a higher prevalence of breastfeeding in the first hour of life among mothers with previous breastfeeding experience (PR = 1.47, 95% CI 1.04–2.07). Difficulty initiating breastfeeding in the birth room was more prevalent among mothers who did not receive breastfeeding guidance during antenatal care (PR = 2.83, 95% CI 1.43–4.32) and those without previous breastfeeding experience (PR = 2.49, 95% CI 1.24–6.45).

**Conclusion:**

These findings highlight the importance of adequate professional guidance, especially for primiparous mothers.

## Introduction

Breastfeeding, as well established in the literature, offers numerous advantages to maternal health, such as some complex complications, like reduced risk of postpartum hemorrhage, prevention of carcinomas (breast and ovary) and even postnatal depression (1, 2). Regarding infant health, besides promoting an optimal nutrition to the baby, the breast milk boosts the immune system against infections and allergies, reduces inflammation activities at postpartum and contributes to mother-child bonding (1–3).

In order to ensure that these benefits are attained, the World Health Organization (WHO) recommends exclusive breastfeeding during the first six months of life and it's also recommended that the mother continues it until the child is two years or older, with complementation in addition (4). Meanwhile, less than half (44%) of infants of the world were exclusively breastfed up to 6 months in 2020 (5). In Brazil, the numbers are similar (45,7%) and indicated a mean duration of 3 months for exclusive breastfeeding and almost 16 months for not exclusive breastfeeding (6). The WHO set a 2030 global target that 70% of infants will be exclusively breastfed up to 6 months of age (7).

Breastfeeding practice can be associated with factors such as previous experience or professional guidance in antenatal and postpartum care, use of artificial nipples, short maternal leave, and breastfeeding complications. The early initiation of breastfeeding, through its practice in the first hour after delivery, is a favorable factor for maternal-child health (1, 8, 9).

The early initiation of breastfeeding reduces the risk of neonatal mortality, and this practice can be favored by antenatal professional guidance and reinforced by the maternity team. Breastfeeding in the first hour of life (BFHL) is highly recommended, especially because it can promote breastfeeding for an extended period of time (10). Therefore, BFHL is one of the steps for the accreditation of institutions by the Baby-Friendly Hospital Initiative (8, 9, 11).

In order to outline effective strategies and promote the BFHL, it is necessary to understand about the wide network that involves the factors associated with this practice and what are the main maternal difficulties in relation to this practice. In this context, this study aims to evaluate the protective factors associated with the early initiation of breastfeeding.

## Materials and methods

This is a cross-sectional study conducted with healthy nursing mothers in the immediate postpartum period (1–10 days after delivery), without age restriction. The data collection was between August and December 2015 in a public maternity hospital located in a Brazilian metropolis (Belo Horizonte, Minas Gerais, 330.9 km², 2,350,564 inhabitants). A minimum sample size of 50 participants was estimated based on BFHL prevalence from a previous study (12), adopting a 95% confidence interval (CI), 5% error, descriptive statistics formulas and finite population (13).

Information on age, education, marital status, occupation, parity, per capita income, gestational age at birth, type of delivery, birth weight and breastfeeding practice were collected through a structured questionnaire and applied up to 48 h postpartum. The interviews lasted about 20 min with each woman and were limited to the questions present in the questionnaire developed for the present study.

The nursing mothers were categorized according to age: adolescents (≤18 years) and adults (>18 years). Gestational age was categorized as preterm (<37 weeks) and term (≥37 weeks) (14).

Birth weight was obtained through medical records and classified according to the criteria of the World Health Organization (15): low (<2,500 g), insufficient (2,500–2,999 g), adequate (3,000–3,999 g) and excessive (>4,000 g).

The nursing women were asked questions related to breastfeeding guidance received in antenatal and postpartum care (yes or no), practice of exclusive breastfeeding—if no other liquid besides breast milk was offered to the child until maternal discharge (yes or no), BFHL (yes or no), difficulty in initiating breastfeeding in the birth room (yes or no), nipple pain during breastfeeding (yes or no) and previous breastfeeding experience (yes or no). The reasons not to practice BFHL and difficulties in initiating breastfeeding were also questioned and from the answers, categories were created according to the most prevalent topics.The mothers did not receive postnatal breastfeeding support from the hospital health team after hospital discharge.

A database was built with the help of the Epi InfoTM 3.5.1 program through double typing, and analyses were performed using the Statistical Package for Social Sciences (SPSS) software (version 19.0) and Stata® (version 14.0). A significance level of 5% was adopted for all the analyses. Kolmogorov-Smirnov test was used to determine if the variables follow a normal distribution. Descriptive analysis was performed by calculating the frequency and mean ± standard deviation. For univariate analysis, Chi-square and Fisher's exact tests were used to estimate the association of the outcomes, “BFHL” and “difficulty initiating breastfeeding in the birth room”, with the variables of the study. For explanatory variables with more than two categories, the Bonferroni correction was applied.

The multivariable models (Poisson regression) included the variables with *p* < 0.20 in the univariate analysis. The stepwise backward method was used to insert the variables, and the goodness-of-fit test was used to adjust the final model. For the variables that presented collinearity, the one with the lowest *p* value in the univariate analysis was maintained.

This study was conducted in accordance with the Helsinki Declaration, and all procedures involving human subjects were approved by the Research Ethics Committee of Federal University of Minas Gerais, under number 52537215.5.0000.5149. The women received oral explanations about the study and a written informed consent was obtained from all participants (for participants under 18 years of age, the legal guardian signed the consent form).

## Results

A total of 104 nursing mothers were evaluated, with a mean age of 25.4 ± 6.8 years, 77.9% (*n* = 81) were adults, 62.1% (*n* = 64) married/stable union, and 52.4% (*n* = 54) had paid work. Most of the deliveries were vaginal (72.1%) and term (79.4%). Further information on the characteristics of the sample is described in Table 1.

**Table 1:** Characteristics of the sample according to the practice of breastfeeding in the first hour of life and difficulty in initiating breastfeeding in the birth room.

Categories	Total	Breastfeeding in the first hour of life			Difficulty in initiating breastfeeding		
	% (*n*)	*N*	Yes	*p* *	*N*	Yes	*p* *
		% (*n*)	% (*n*)		% (*n*)	% (*n*)	
Age							
Adolescent	22.1 (23)	28.8 (13)	16.9 (10)	0.112	78.9 (45)	76.7 (33)	0.490
Adult	77.9 (81)	71.1 (32)	83.1 (49)		21.1 (12)	23.3 (10)	
Occupation							
Paid employment	52.4 (54)	52.3 (23)	52.5 (31)	0.171	48.2 (27)	55.8 (24)	0.607
Student	7.8 (8)	13.6 (6)	3.4 (2)		7.1 (4)	9.3 (4)	
Homemaker	30.1 (31)	22.7 (10)	22.7 (10)		35.7 (20)	23.3 (10)	
No remuneration	9.7 (10)	11.4 (5)	11.4 (5)		8.9 (5)	11.6 (5)	
Education							
Primary	33.7 (35)	31.1 (14)	35.6 (21)	0.429	38.6 (22)	25.6 (11)	0.322
High school	58.7 (61)	64.4 (29)	54.2 (32)		52.6 (30)	67.4 (29)	
Higher education	7.6 (8)	4.4 (2)	10.2 (6)		8.8 (5)	7.0 (3)	
Marital status							
Single	37.9 (39)	42.2 (19)	34.5 (20)	0.422	33.3 (19)	44.2 (19)	0.268
Married/Stable union	62.1 (64)	57.8 (26)	65.5 (38)		66.7 (38)	55.8 (24)	
Per capita income^†^							
≤0.5 minimum wage	42.4 (42)	34.9 (15)	48.2 (27)	0.130	58.2 (32)	22.5 (9)	**0** **.** **001**
>0.5 minimum wage	57.6 (57)	65.1 (28)	51.8 (29)		41.8 (23)	77.5 (31)	
Parity							
Primiparous	50.0 (52)	62.2 (28)	40.7 (24)	**0** **.** **024**	35.1 (20)	69.8 (30)	**0** **.** **001**
Multiparous	50.0 (52)	37.8 (17)	59.3 (35)		64.9 (37)	30.2 (13)	
Gestational age							
Preterm	20.6 (21)	25.6 (11)	16.9 (10)	0.207	12.5 (7)	31.0 (13)	**0** **.** **024**
Term	79.4 (81)	74.4 (32)	83.1 (49)		87.5 (49)	69.0 (29)	
Type of delivery							
Vaginal	72.1 (75)	71.1 (32)	72.9 (43)	0.507	73.7 (42)	72.1 (31)	0.518
Cesarean section	27.9 (29)	28.9 (13)	27.1 (16)		26.3 (15)	27.9 (12)	
Birth weight							
Insufficient/Low weight	43.6 (44)	51.2 (22)	37.9 (22)	**0** **.** **009**	26.3 (15)	22.5 (9)	0.198
Adequate	51.5 (52)	46.5 (20)	55.2 (32)		52.6 (30)	50.0 (20)	
Excessive	4.9 (5)	2.3 (1)	6.9 (4)		7.0 (4)	2.5 (1)	
Breastfeeding guidance (antenatal)							
No	70.9 (73)	79.5 (35)	64.4 (38)	0.094	59.6 (34)	88.1 (37)	**0** **.** **003**
Yes	29.1 (30)	20.5 (9)	35.6 (21)		40.4 (23)	11.9 (5)	
Breastfeeding guidance (postpartum)							
No	76.9 (80)	20.0 (9)	25.4 (15)	0.515	33.3 (19)	9.3 (4)	**0** **.** **007**
Yes	23.1 (24)	80.0 (36)	74.6 (44)		66.7 (38)	90.7 (39)	
Nipple pain during breastfeeding							
No	49.0 (49)	45.2 (19)	51.7 (30)	0.331	50.9 (29)	46.5 (20)	0.409
Yes	51.0 (51)	54.8 (23)	48.3 (28)		49.1 (28)	53.3 (23)	
Previous breastfeeding experience							
No	51.9 (54)	64.4 (29)	42.4 (25)	**0** **.** **026**	36.8 (21)	72.1 (31)	**0** **.** **001**
Yes	48.1 (50)	35.6 (16)	57.6 (34)		63.2 (36)	27.9 (12)	

* *P* value Chi-square test. *P* values in bold are statistically significant.

^†^ Minimum wage: R$ 788.00 (2015).

In relation to breastfeeding, 89.4% (*n* = 93) reported exclusive breastfeeding, 56.7% (*n* = 59) BFHL and 43.0% (*n* = 43) difficulties initiating breastfeeding in the birth room.

The reasons presented by the nursing mother for not BFHL (Supplementary Table S1) were health problems of the mother (27.3%) and the baby (25.0%), incorrect baby latch (18.2%), delayed onset of lactation (15.9%) and absence of antenatal professional guidance (13.6%). Regarding the reasons for difficulty initiating breastfeeding in the birth room (Supplementary Table S1), the following were reported: incorrect baby latch (59.5%), delayed onset of lactation (21.4%) and nipple pain during breastfeeding (19.1%).

The univariate analysis results are presented in Table 1. BFHL was associated with parity (*p* = 0.024), breastfeeding experience (*p* = 0.026) and birth weight (*p* = 0.009). Difficulty in initiating breastfeeding in the birth room was associated with per capita income (*p* = 0.001), parity (*p* = 0.001), gestational age (*p* = 0.024), breastfeeding experience (*p* = 0.001), postnatal (*p* = 0.007) and antenatal breastfeeding guidance (*p* = 0.003).

The results of the multivariate analysis are presented in Table 2. The prevalence of BFHL was 1.47 times higher among nursing mothers with previous breastfeeding experience (*p* = 0.03). In addition, the prevalence of difficulty initiating breastfeeding in the birth room was 2.83 times higher among women who did not receive practice guidance of breastfeeding in antenatal care (*p* = 0.013) and 2.49 times higher among those who did not present breastfeeding experience (*p* = 0.001).

**Table 2:** Poisson regression analysis for the association between the variables and the outcomes “breastfeeding in the first hour of life” and “difficulty in initiating breastfeeding in the birth room".

Outcome	Variable	PR	CI 95%	*p*
Breastfeeding in the first hour of life	Previous breastfeeding experience			0.03
	No	1.00	–	
	Yes	1.47	1.04–2.07	
Difficulty in initiating breastfeeding in the birth room	Breastfeeding guidance in antenatal care			0.013
	No	2.83	1.24–6.45	
	Yes	1.00	–	
	Previous breastfeeding experience			0.001
	No	2.49	1.43–4.32	
	Yes	1.00	–	

PR, prevalence ratio; CI, confidence interval.

## Discussion

The prevalence of BFHL in the present study (56.7%) was higher than in other cross-sectional study (31%) conducted with 320 nursing mothers of another Brazilian metropolis (16). On the other hand, it was similar to the Brazilian prevalence (45,8%) as described in a national survey (4). The WHO considers breastfeeding in the first hour an indicator of excellence, classifying the percentages between zero to 29% as “very bad”, 30 to 49% “bad”, 50 to 89% “good” and from 90 to 100 as very good (17). Although the prevalence rates found in these studies were classified as “good”, the encouragement and implementation of breastfeeding in the first hour are necessary in a progressive way to ensure the benefits of the practice to the mother and child for a longer period of time.

It should be noted that the current study was carried out in a maternity that is not accredited by the Baby-Friendly Hospital Initiative, which has as one of its basic steps the promotion of BFHL, which indicates that the prevalence classified as “good” could reach even better ratings due to the efforts and work achieved by the initiative. Previous studies show that BFHL is associated with a number of positive health outcomes such as maternal-infant bond, strengthening the immune system, preventing the early use of infant formula (18) and reducing the risk of neonatal mortality (1, 19).

Among the most quoted causes that prevent BFHL, cesarean delivery was the main one. In contrast to the previous studies (20, 21), BFHL was not, in this study, significantly associated with cesarean birth because another factor, such as the previous breastfeeding experience, had a greater impact to explain the outcome. Cesarean section delays early initiation of breastfeeding, and this delay is attributed majorly to the practice of separating the newborn from mothers immediately after cesarean childbirth (20). However, mothers who had previous breastfeeding experience generally know that the practice of breastfeeding in the first hour of life is important to promote the child's health and reduce neonatal mortality.

In the present study, both BFHL and the difficulty initiating breastfeeding in the birth room were associated with previous breastfeeding experience. Corroborating these findings, other studies found an association between BFHL and previous experience with breastfeeding (22), and with the number of children (23).

The variables, parity and previous breastfeeding experience, may be correlated, considering that multiparous women are more likely to have had previous contact with breastfeeding, and women with their first child are at a greater risk of presenting difficulty initiating breastfeeding (23). In this context, primiparous mothers should be especially targeted by professional breastfeeding guidance.

Antenatal breastfeeding guidance was a protective factor for difficulty initiating breastfeeding in the birth room, and the guidelines offered during prenatal care can facilitate this process (22). A previous study showed that the chance of BFHL decreases by 28% if antenatal care is not properly performed (23).

Breastfeeding guidance in antenatal care and after delivery is very important to establish BFHL and to increase the chance of breastfeeding continuation after hospital discharge. A meta-analysis performed by Rollins et al. (9) found that individual counseling and immediate postpartum breastfeeding support increased the prevalence of any type of breastfeeding until six months of age by 66.0% and exclusive breastfeeding by 49.0%. In addition, interventions that combined pre and postnatal counseling were more effective than those which target only one specific period (9).

The present study has some limitations, such as the cross-sectional design which does not allow the establishment of causal relationships, and sample homogeneity associated with the study's setting being a single maternity hospital.

Despite the aforementioned limitations, the study presents a low memory bias in the evaluation of breastfeeding initiation, considering that the questionnaires were applied in the immediate postpartum period. In addition, few studies in the literature have described factors associated with the difficulty of initiating breastfeeding in the birth room. It is important to emphasize that this study can guide effective actions to help promoting the early breastfeeding initiation.

In summary, breastfeeding guidance during the antenatal care favored a lower prevalence of difficulty initiating breastfeeding in the birth room. Previous breastfeeding experience was a protective factor for both outcomes of the study. Thus, it is clear the importance of promoting adequate antenatal care, including guidance on breastfeeding, with special attention to those without previous breastfeeding experience. Finally, it is important to encourage the early breastfeeding establishment in order to increase the chance of breastfeeding success after hospital discharge.

## Data Availability

The original contributions presented in the study are included in the article/**Supplementary Material**, further inquiries can be directed to the corresponding author.
