# Supplementary material for: Protective factors for early initiation of breastfeeding among Brazilian nursing mothers
Source: Front Pediatr. 2023 Jun 9;11:1203575. doi: 10.3389/fped.2023.1203575 (PMC10288142; doi:10.3389/fped.2023.1203575)
Supplement: Supplementary file 1 [file Table1.docx]

Protective factors for early initiation of breastfeeding among Brazilian nursing mothers

Giovana Gaglianone Lemos^1^, Taciana Maia de Sousa^2^, Rafaela Cristina Vieira e Souza^3*^, Larissa Bueno Ferreira^4^, Cristianny Miranda^2^, Luana Caroline dos Santos^2^.

^1^Nutritionist. Nutrition Department, Universidade Federal de Minas Gerais, Belo Horizonte, Minas Gerais, Brazil.

^2^Ph.D. Nutrition Department, Universidade Federal de Minas Gerais, Belo Horizonte, Minas Gerais, Brazil.

^3^M.D. Nutrition Department, Universidade Federal de Minas Gerais, Belo Horizonte, Minas Gerais, Brazil.

^4^Ph.D. Nutrition Department, Universidade de Brasília, Brasília, Distrito Federal, Brazil.

*** Correspondence:**Rafaela Cristina Vieira e Souza
rafasouzacec@gmail.com

**Table S1** - Reasons presented by the nursing mothers for not breastfeeding in the first hour of life and difficulty in initiating breastfeeding in the birth room

| **Reasons for not Breastfeeding in the first hour of life** | **%** |
| --- | --- |
| Health problems of the mother | 27.3 |
| Health problems of the baby | 25.0 |
| Incorrect baby latch | 18.2 |
| Late lactation | 15.9 |
| Absence of professional guidance | 13.6 |
| **Reasons for difficulty in initiating breastfeeding in the birth room** | **%** |
| Incorrect Baby Latch | 59.5 |
| Late Lactation | 21.4 |
| Nipple Pain During Breastfeeding | 19.1 |
